# First complete mitochondrial genome of *Antheraea pernyi* Guérin-Méneville 1855 (Lepidoptera: Saturniidae) from North Korea

**DOI:** 10.1080/23802359.2021.2002216

**Published:** 2021-11-23

**Authors:** Shu-Wei Sun, En-Guang Tan, Yan-Qun Liu

**Affiliations:** a College of Agronomy, Eastern Liaoning University, Dandong, PR China; b College of Bioscience and Biotechnology, Shenyang Agricultural University, Shenyang, PR China

**Keywords:** *Antheraea pernyi*, Dingzhou_1, mitochondrial genome

## Abstract

This study reports the first complete mitochondrial genome of *Antheraea pernyi* Guérin-Méneville 1855 (Lepidoptera: Saturniidae) strain Dingzhou_1, a silkworm resource serving silk production in North Korea. The mitochondrial genome is circular with 15,573 bp in length encoding 37 typical mitochondrial genes (13 protein-coding genes, two ribosomal *RNA* genes, and 22 transfer *RNA* genes). The 553-bp A + T-rich region harbors a repeat region composed of 6 ∼ 38 bp tandem repeat units, as found in other known inbred strains from Chinese populations. The phylogenetic analysis clustered Dingzhou_1 from North Korea together with Chinese Liaoning population, suggesting that oak silkworm in North Korea might be introduced from her neighbor China Liaoning.

The Chinese oak silkworm, *Antheraea pernyi* Guérin-Méneville 1855 (Lepidoptera: Saturniidae), is one of the most important silkworm resources to produce silk in North Korea. North Pyongan Province of North Korea, near its border with China, is one of the traditional production areas for Chinese oak silkworm. It has been suggested that this importantly economic insect might be introduced into North Korea from China in the middle 18th century based on the culture exchange between the two countries (Sericultural Institute of Liaoning Province 1994); this deduction needs direct genetic evidence to support it. However, the genetic information regarding this important silkworm resource in North Korea remains severely limited (Chen et al. 2012). In this study, we report the first complete mitochondrial genome of *A. pernyi* from North Korea that will help understand the origin of oak silkworm in North Korea.

The strain Dingzhou_1 used in this study was developed by the Chongju Seed Station in North Pyongan Province of North Korea. This strain has been successively preserved at the Sericultural Institute of Liaoning (N 40°28′16.45′′; E 124°04′20.30′′), Fengcheng, China since introduction in 1980 (Sericultural Institute of Liaoning Province 1994). The specimens of first-instar larvae preserved in 95% ethanol were deposited at the Department of Sericulture, Shenyang Agricultural University, China (https://www.syau.edu.cn/, Dr. Yan-Qun Liu, liuyanqun@syau.edu.cn) under the voucher number OAK_SILKWORM_DINGZHOU_01. A single larva was used to extract the total genomic DNA. Two overlapping long fragments of mitochondrial genome were amplified as described (Li et al. 2021), and subjected to sequence determination on the Illumina PE 150 platform (Illumina Inc, San Diego, CA). Genome assembly was performed on the Galaxy web server at usegalaxy.org (https://usegalaxy.eu/) (Jalili et al. 2020).

The whole mitochondrial genome of *A. pernyi* strain Dingzhou_1 is 15,573 bp in length (GenBank accession number MW940851). It contains 13 protein-coding genes, 22 *tRNA* genes, two ribosomal *RNA* genes, and a non-coding A + T-rich region, exhibiting an identical genomic architecture with known *Antheraea* species, such as Qinghuang_1, the first modern improved strain of *A. pernyi* (Li et al. 2021). The start and stop codons identified in this genome are also identical to that in Qinghuang_1. The A + T-rich region in the mitochondrial genome of Dingzhou_1 spans 553 bp in size harboring a repeat region composed of 6 ∼ 38 bp tandem repeat units, as found in other known inbred strains from Chinese populations (Chen et al. 2012). The 20-bp poly-T stretch preceded by the ATAGA motif identified in this genome is the largest one among known *A. pernyi* mitochondrial genomes. We identified one SNP (G/C) and one indel (–/T) in the A + T-rich region between Dingzhou_1 determined in this study (MW940851) and an individual of Dingzhou_1 (JQ028689) reported previously, and one indel (–/T) between Dingzhou_1 (MW940851) and 64 (JQ028691), indicating an extremely low degree of sequence divergence in the North Korean population of oak silkworm.

To explore the intraspecific phylogenetic position of Dingzhou_1 that was from North Korea, a phylogenetic tree was reconstructed based on the nucleotide sequences of eight whole mitochondrial genomes of *A. pernyi* available to date (Figure 1). An *Antheraea* species (*A. assamensis*) and a non-*Antheraea* species (*Eriogyna pyretorum*) served as outgroups. Phylogenetic tree was built using maximum likelihood method under GTR + G + I model in MEGA X (Kumar et al. 2018). In the phylogenetic tree obtained, two clades were recovered for eight *A. pernyi* strains: one contained Dingzhou_1, Qing_6, and Qinghuang_1, and the other included the remaining five strains. According to the breeding records (Sericultural Institute of Liaoning Province 1994), Qing_1 and Qinghuang_1 were derived from Chinese Liaoning population ‘Aiyang’, and the other five strains came from Chinese Henan population ‘Lushan’. The phylogenetic analysis indicated that Dingzhou_1 from North Korea has a close relationship with Chinese Liaoning population, which suggested that oak silkworm in North Korea might be introduced from her neighbor China Liaoning. Further in-depth analysis for understanding the origin of oak silkworm resources in North Korean should be required in near future using more mitochondrial genomes of the strains from Chinese populations.

**Figure 1.**
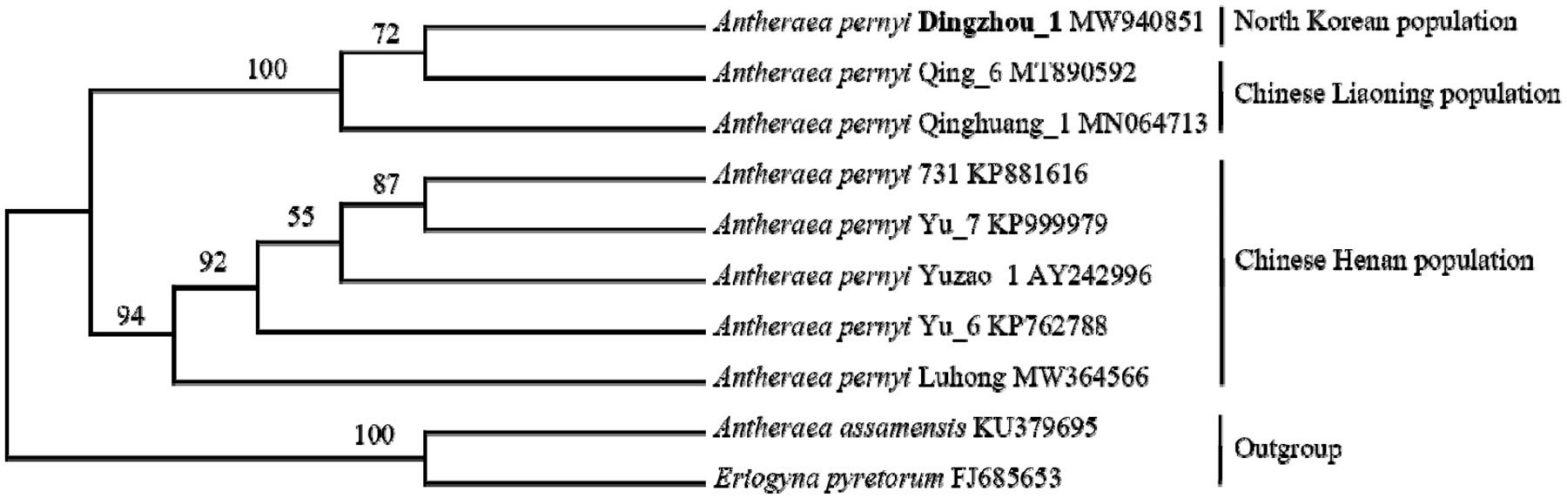
Phylogenetic tree inferred from nucleotide sequences of whole mitochondrial genomes with maximum likelihood method. The GTR + G + I model was selected according to the Akaike information criterion in MEGA X. The confidence values were evaluated *via* a bootstrap test with 1000 replications. GenBank accession numbers are listed following the name of each species or strain.

## Data Availability

The genome sequence data that support the findings of this study are openly available in GenBank of NCBI at (https://www.ncbi.nlm.nih.gov/) under the accession No. MW940851. The associated BioProject, SRA, and bio-sample numbers are PRJNA764361, SRR16079440, and SAMN21848561, respectively.
